# Characterizing impulsivity in individuals with methamphetamine and methcathinone use disorders

**DOI:** 10.3389/fpsyt.2024.1416342

**Published:** 2024-10-16

**Authors:** Jie Yin, Xinyu Cheng, Chendi Zhou, Lin Xu, Bo Yang, Ti-Fei Yuan

**Affiliations:** ^1^ School of Psychology, Beijing Sport University, Beijing, China; ^2^ Laboratory of Sports Stress and Adaptation of General Administration of Sport, Beijing Sport University, Beijing, China; ^3^ Shanghai Key Laboratory of Psychotic Disorders, Brain Health Institute, National Center for Mental Disorders, Shanghai Mental Health Center, Shanghai Jiao Tong University School of Medicine, Shanghai, China; ^4^ School of Sociology, China University of Political Science and Law, Beijing, China; ^5^ Co-innovation Center of Neuroregeneration, Nantong University, Nantong, Jiangsu, China

**Keywords:** methcathinone, methamphetamine, impulsivity, network-based analysis, sensation seeking, delay discounting

## Abstract

**Background:**

Individuals with substance use disorder (SUD) are characterized by loss of control in drug use, such as increased impulsivity. Methamphetamine and methcathinone are psychostimulants, the use of which is accompanied by a high level of impulsivity. Whether individuals with methamphetamine use disorder (MUD) and methcathinone use disorder (MCUD) differ in different aspects of impulsivity is unclear.

**Methods:**

We investigated impulsivity traits and behaviors in individuals with MUD and MCUD. The Barratt Impulsiveness Scale (BIS), Sensation Seeking Scale (SSS), and delay discounting task (DDT) were assessed in individuals with MUD and MCUD and in healthy controls (HCs); then, we performed network-based analysis and computational modeling to understand the potential differences among the three groups.

**Results:**

MUD subjects scored higher than MCUD subjects in terms of motor impulsivity, nonplanning impulsivity, and total BIS scores. The network analysis revealed that there were no significant differences between MUD and MCUD subjects in any centrality indices. The discount rate of MUD and MCUD subjects was significantly greater than that of HCs, whereas there was no difference in the discount rate between the two addiction groups.

**Conclusions:**

These findings suggest that MUD and MCUD participants differ in impulsivity traits but not in impulsive behaviors, implying that impulsive traits and behaviors represent different aspects of impulsivity.

## 1 Introduction

Numerous studies have consistently shown that substance abuse is associated with impairments in cognitive function, such as attention (1), decision making (19), inhibitory control (2, 3), and structural and functional abnormalities in the brain (4, 5). Substance addiction is a periodic or chronic toxic state caused by the continuous use of one substance, and its defining characteristic is compulsive, out-of-control drug use despite serious negative consequences (6). Individuals with substance use disorder (SUD) exhibit characteristics of impulsivity, and impulsive behavior is closely linked to drug use (7, 8). Impulsivity has been defined as “a predisposition toward rapid, unplanned reactions to internal or external stimuli without regard to the negative consequences of these reactions” (9). It is a multidimensional construct comprising different aspects (7) and is not only an inherent part of standard individual differences in personality but also intersects with more dysfunctional and pathological behaviors (10). Researchers have proposed that impulsivity may serve as both a consequence and a determinant of drug use (11).

Methcathinone is a third-generation drug or a new psychoactive substance that is commonly known as “zombie drug” and is an analog of amphetamine (12). Methcathinone abuse can cause cognitive impairment in users (13, 14). Both methamphetamine and methcathinone have similar molecular structures, and both are psychostimulants (15). However, compared with methamphetamine, methcathinone is more hydrophilic and less likely to penetrate the blood‒brain barrier; therefore, higher doses are required to achieve similar effects (16). The intravenous administration of these two drugs has different effects on the brain (54).

Long-term exposure to methamphetamine increases impulsivity in rats (17). Individuals with methamphetamine use disorder (MUD) have higher impulsivity scores than healthy controls (18), show an impulsive decision-making pattern, and tend to prefer small immediate rewards over large, delayed rewards when faced with a choice (19). Brain imaging studies have revealed that the long-term chronic use of methamphetamine can lead to functional disorders in the frontal lobe (20), and frontal lobe damage is significantly related to impulsivity (21).

Previous studies have also suggested that methcathinone abuse is associated with impulsivity. Methcathinone use can induce violent and aggressive behavior (22). Individuals with methcathinone use disorder (MCUD) exhibit personality changes, including increased aggression and destructiveness (23). Studies have shown that aggression is associated with impulsivity (24). Individuals with MCUD have high levels of impulsivity (25). These individuals also have impaired frontal executive function (13, 26), suggesting that they have inhibitory control dysfunction, which means that they cannot suppress impulsive behaviors including drug seeking.

At present, many studies related to methamphetamine exist, whereas few studies have focused on methcathinone. Do they have similar effects on impulsivity? In other words, are there differences in impulsivity between abusers of these two drugs? There are a few studies related to this issue, one of which reported that the impulsivity of methamphetamine addicts was significantly greater than that of methcathinone addicts (27). However, whether they differ in other aspects of impulsivity is unclear. Since the effects of methcathinone use on individuals’ impulsivity have received little attention in the literature, we focused on this group first. Our previous study revealed that MCUD subjects had deficits in problem-solving ability, a high-level executive function, especially under high task difficulty load conditions (28), indicating that individuals with MCUD exhibit abnormal inhibitory control. Therefore, we sought to understand the differences in impulsivity between MUD participants and MCUD participants and whether knowledge of these differences would facilitate precision interventions for different drug users.

Therefore, two separate studies were conducted to assess the differences in impulsivity between individuals with MUD and MCUD. In study 1, the Barratt Impulsiveness Scale (BIS) (29) was used to test the differences in impulsive personality traits between the two addiction groups. Researchers have reported that impulsivity and sensation seeking are correlated dimensions of personality (30). Zuckerman combined sensation seeking and impulsivity into a supertrait called impulsive sensation seeking (31). Sensation seeking is an important personality trait that affects adolescent substance use, and it is closely related to addictive behaviors (32). Therefore, we also tested differences in sensation seeking between the two drug groups via the Sensation Seeking Scale (SSS) (33). Additionally, given the close relationship between impulsivity and sensation seeking, we employed network-based analysis to construct trait impulsivity and sensation seeking networks to characterize the interactions between the two different traits among the three groups. In study 2, the delay discounting task (DDT) (34) was used to investigate the differences in impulsive behaviors between the two addiction groups. According to previous studies, one of the characteristic behaviors of addicted individuals is their inability to adopt adaptive strategies to achieve future positive outcomes. They tend to choose immediate rewards (e.g., drug use) rather than restraining their desires to gain long-term benefits (e.g., good health) (35, 36). Therefore, we believe that impulsive decision-making is an important reflection of their actual behavior.

On the basis of previous studies, we hypothesized that MUD subjects would have higher scores on the impulsive scale and sensation seeking than MCUD subjects and that MUD subjects would exhibit higher levels of impulsive behavior than MCUD subjects.

## 2 Methods

### 2.1 Participants

Studies 1 and 2 were approved by the ethics committee of China University of Political Science and Law. All participants in studies 1 and 2 had normal or corrected-to-normal vision, and they all provided informed consent for their voluntary involvement in the study. All procedures adhered to the principles outlined in the Declaration of Helsinki. The demographic information is shown in **Table 1**.

**Table 1:** Demographic characteristics of the three groups in studies 1 and 2.

		MUD	MCUD	HC	*F*(*t*)	*p*	*η*²
		M (SD)	M (SD)	M (SD)			
Study 1	Age	33.31 (7.44)	37.48 (6.85)	33.28 (10.59)	16.7	<0.001	0.053
	Education(years)	8.98 (3.10)	8.60 (2.14)	11.24 (4.01)	40.25	<0.001	0.119
	Years of drug use	6.40 (4.60)	3.70 (3.02)	/	6.94	<0.001	0.697
	Dosage of drug use (g/one time)	0.74 (0.68)	0.79 (0.62)	/	0.758	0.449	/
Study 2	Age	32.32 (6.53)	35.43 (6.79)	31.79 (10.01)	2.27	0.108	/
	Education(years)	10.00 (2.95)	9.49 (2.31)	11.03 (3.23)	2.86	0.062	/
	Years of drug use	6.56 (4.57)	4.14 (2.77)	/	2.76	0.007	0.640
	Dosage of drug use (g/one time)	0.87 (0.58)	0.90 (0.62)	/	0.22	0.826	/

#### 2.1.1 Subjects of study 1

A total of 206 MCUD participants (age range: 22–51 years) and 198 MUD participants (age range: 19–56 years) were recruited from the Compulsory Detoxification Center for men in Changzhi, Shanxi Province, China. They were all male, and all had positive results on the urine methamphetamine or methcathinone tests before they entered the center. They had been abstinent for 2–4 months prior to the study. The primary route of administration was snorting, and no one had injected drugs. Their self-reported frequency of drug use was once a week or more 6 months before abstinence. The inclusion criteria were as follows: primary diagnosis of MUD or MCUD, no concurrent neurological or psychiatric disorders, no ongoing psychiatric medications, and no hallucinations or acute withdrawal symptoms. These diagnoses were confirmed by a senior psychiatrist via the Structured Clinical Interview for DSM-5. The police officers at the center communicated with these eligible subjects and asked if they were willing to participate in the study. They were told the study procedure and provided signed informed consent before enrollment in the study.

In total, 210 healthy male controls (HCs) were recruited via advertisements in the local communities of Changzhi, Nanjing, and Beijing. The control group mainly consisted of security guards, drivers, and factory workers. They were also told the study procedure and provided signed informed consent before enrollment in the study. A total of 17 questionnaires were excluded because of careless answers, missing answers, or other reasons. A sample of 193 participants was ultimately included in the analyses (age range: 18–57 years).

#### 2.1.2 Subjects of study 2

A total of 38 MUD participants (age range: 21–48 years) and 38 MCUD participants (age range: 22–46 years) were also recruited from the Compulsory Detoxification Center for men in Changzhi, Shanxi Province. The duration of abstinence of these participants was 2–4 months prior to the study. The primary route of administration was snorting, and no one had injected drugs. The inclusion criteria for the groups were as described in study 1. A total of 40 nonaddicted healthy control subjects (age range: 18–51 years) were recruited from Changzhi and Beijing. The two addiction groups and the control group were matched in terms of age and length of education. Moreover, 68 subjects in the addiction groups in study 2 also participated in study 1. All of the participants in study 2 were also male.

### 2.2 Study procedure and task materials

#### 2.2.1 Study 1

The participants in Study 1 were administered questionnaires that included a demographic information questionnaire, the BIS-11, and the SSS.

Barratt Impulsiveness Scale (BIS-11): The Chinese version of this impulsiveness scale, which was translated and revised by the Beijing Psychological Crisis Research and Intervention Center, was used in this study (37). The BIS-11 contains 30 items and is divided into three dimensions: nonplanning, attentional impulsivity, and motor impulsivity. The participants were asked to assess how often each item occurred on a scale ranging from 1 (not at all) to 5 (always). If a participant scores high in nonplanning, it means that he or she lacks planning. High scores in attentional impulsivity represent a tendency to make rapid decisions. High scores in motor impulsivity represent a lack of consideration before taking action. The higher the total score is, the stronger the level of impulsivity. Confirmatory factor analysis revealed that the questionnaire had good structural validity: χ²/*df* = 3.59, GFI = 0.86, RMSEA = 0.06. In this study, the Cronbach’s *α* coefficient was 0.927.

Sensation Seeking Scale (SSS): The SSS was first developed by Zuckerman (38). The questionnaire used in this study is the Chinese version of the fifth edition revised by Wang et al. (33). The questionnaire contains 40 items. Each item includes two descriptive sentences. The subjects were asked to choose the one closest to their situation. The questionnaire has four dimensions: thrill and adventure seeking, experience seeking, disinhibition, and boredom susceptibility. Confirmatory factor analysis revealed that the questionnaire had good structural validity: *χ*²/*df* = 2.22, GFI = 0.88, RMSEA = 0.04. In this study, the Cronbach’s *α* coefficient was 0.746.

#### 2.2.2 Study 2

Delay discounting task (DDT): The monetary delay discounting task used in study 2 was administered via a computer. Two types of rewards were presented on the screen: smaller immediate rewards were on the left, and larger delayed rewards were on the right. The subjects were required to make preference judgments between the two hypothetical rewards. The participants were offered two immediate rewards—50 and 100 yuan—as well as eight delayed options spanning from 1 to 360 days—specifically 1, 3, 7, 21, 45, 90, 180, and 360 days. The delayed reward amount was not fixed and varied depending on the subject’s response.

In the task, the order of the immediate reward and the order of the delay time for the same immediate reward were randomized for each subject. The specific amount for each delay was determined on the basis of the subject’s response. If the subject chose the immediate reward in the current trial, then the amount of the delayed reward in the next trial would be increased. If the subject chose a delayed reward, the amount of the delayed reward in the next trial would be reduced.

The subjects were asked to press the “F” key for selecting the immediate rewards and the “J” key for selecting the delayed rewards. After the subjects understood the instructions, they were required to perform three practice trials first and then perform the formal experiment. The formal experiment included 128 trials.

### 2.3 Data analysis

#### 2.3.1 Analysis of demographic characteristics and questionnaires

For study 1, the demographics, BIS scores, and SSS scores among the two addiction groups and the control group were compared using ANOVA. Significant differences were observed in both age and years of education among the three groups; thus, the two variables were treated as covariates in the ANOVA for BIS scores and sensation seeking. *Post hoc* comparisons were also conducted to examine which two groups were different (FDR correction). When the assumption of homogeneity of variance was not satisfied, Brown–Forsythe test and Games–Howell tests for multiple comparisons were used. Independent-sample *t*-tests were used to evaluate the differences in the dosage of drug used between MUDs and MCUDs. A correlation analysis was used to test the correlation between the BIS score and sensation seeking among the three groups. For study 2, the demographics of the three groups and the dosage of drug used were compared between the MUD and MCUD groups, as was the case in study 1. The significance level alpha was set to 0.05 (two-tailed).

#### 2.3.2 Network analysis

Network analysis was conducted in R Studio using the bootnet (version 1.5.3), qgraph (version 1.9.5), and NetworkComparisonTest (version 2.2.1) packages. A Gaussian graphical model (GGM) was employed to construct trait impulsivity and sensation seeking networks for each of the three groups in study 1. In these networks, the three dimensions of the BIS and the four dimensions of the SSS were treated as nodes, with partial correlations between pairs of nodes representing edges. The edges were regularized using the EBICglasso (Extended Bayesian Information Criterion Graphical Lasso, EBICglasso) procedure, which optimizes model sparsity through two key hyperparameters: Lambda (λ), which controls the sparsity of the graphical model, and Gamma (γ), which is set to 0.5 to balance the model’s sensitivity and specificity. Lambda was varied across 100 logarithmically spaced values between λ_max (the maximum value where all edges are zero) and λ_max/100. The extended Bayesian information criterion (EBIC) was calculated for each network, with the graph having the best EBIC selected. To minimize type I errors, edges with small weights were penalized to zero.

The network structure is characterized by network centrality indices, i.e., strength, closeness, and betweenness (39). Centrality measures the importance of a node in determining the network’s structure (40). Strength represents the weighted sum of edges directly connected to a node and measures the importance of a feature in the network. Closeness represents the inverse of the sum of the average shortest path length between a node and all other nodes. It measures the closeness between a feature and other features. Betweenness represents the number of times that the shortest path between any two nodes passes through another node. It measures the importance of the feature in linking to other features. All node centralities were calculated for each of the three networks, and differences in centrality indices (strength, closeness, and betweenness) were compared among the three networks.

The network properties were compared between any two groups using permutation tests with 1,000 iterations (41, 42). The participants in two of these groups were randomly assigned to two groups when the differences between the two groups were compared. The networks were subsequently constructed, estimated, and compared using a bootstrap resampling method, which was repeated 1,000 times to obtain the null distribution of the network differences under the null hypothesis.

#### 2.3.3 Analysis of discount rate (*k*)

The discount rate functions as an indicator of impulsivity, with a higher rate signifying greater impulsivity (34). Delay discounting was estimated by fitting the data to the hyperbolic function equation: *V* = *A*/(1 + *kD*). *A* is the value of the delay reward, and *D* represents the delay days. *V* is the value of amount *A* at delay *D* (in days). *k* stands for the index of delay discounting (discount rate). In this study, *V* was fixed and composed of two amounts (50 and 100 yuan), whereas *D* had eight different numerical values (from 1 day to 360 days). The amount of *A* varied according to the subject’s choices. In this way, the parameter *k* was calculated through nonlinear regression. The index of best fit was *R* ^2^ = 0.94, indicating an ideal fitting effect. Since the distribution of the *k* value did not conform to the normal distribution, a natural logarithmic transformation was applied to the *k* value, resulting in *k*′. The *k*′ value of the two addiction groups and the control group in study 2 were subsequently compared via ANOVA.

## 3 Results

### 3.1 Subject demographic characteristics and history of drug use

The demographics and comparisons among the different groups are shown in **Table 1**. In study 1, significant differences were observed in the age and years of education among the three groups. *Post hoc* analysis showed that the MCUD group was significantly older than both the MUD group (*p* < 0.001) and the HCs (*p* < 0.001). However, age was not different between the MUD group and the HCs. *Post hoc* analysis also revealed that the HC group had significantly more years of education than the two addiction groups (*p* < 0.001), whereas no significant difference was detected between the two addiction groups. Compared with the MCUD group, the MUD group had significantly more years of drug use. No significant difference was found in the one-time dosage of drug used between the /MUD and MCUD groups.

In study 2, as shown in **Table 1**, no significant differences were observed among the three groups in terms of age or years of education. There was a significant difference in the number of years of drug use between the two addiction groups, but not in the one-time dosage of drug used.

### 3.2 Differences in the BIS

Owing to the differences among the three groups in terms of age and years of education, these two variables were included as covariates in the ANOVA. The analyses revealed that (as shown in **Table 2**) significant differences were found among these three groups in nonplanning impulsivity, attentional impulsivity, motor impulsivity, and the total score.

**Table 2:** Comparison of impulsivity and sensation seeking scores among MUD, MCUD, and HC.

	MUD	MCUD	HC	*F*	*η*²
	M (SD)	M (SD)	M (SD)		
AI	27.47 (7.30)	26.80 (7.12)	22.92 (5.88)	18.58***	0.059
MI	25.56 (8.47)	23.29 (7.29)	22.02 (7.38)	8.22***	0.027
NPI	30.14 (9.12)	28.12 (7.98)	21.81 (7.03)	45.97***	0.134
TI	83.17 (20.54)	78.21 (17.33)	66.75 (16.55)	32.86***	0.100
TAS	5.74 (2.47)	5.34 (2.48)	4.68 (2.51)	8.91***	0.029
ES	3.46 (1.68)	2.66 (1.55)	3.60 (2.02)	11.15***	0.036
DIS	5.09 (2.54)	3.55 (2.01)	2.95 (1.78)	49.61***	0.144
BS	2.30 (1.69)	2.03 (1.39)	2.35 (1.86)	1.46	0.005
TS	16.59 (5.18)	13.59 (4.75)	13.58 (5.94)	17.20***	0.055

AI, attentional impulsivity; MI, motor impulsivity; NPI, nonplanning impulsivity; TI, total impulsivity; TAS, thrill and adventure seeking; ES, experience seeking; DIS, disinhibition; BS, boredom susceptibility; TS, total SSS score.

*p < 0.05; **p < 0.01; ***p < 0.001.

**Figure 1:**
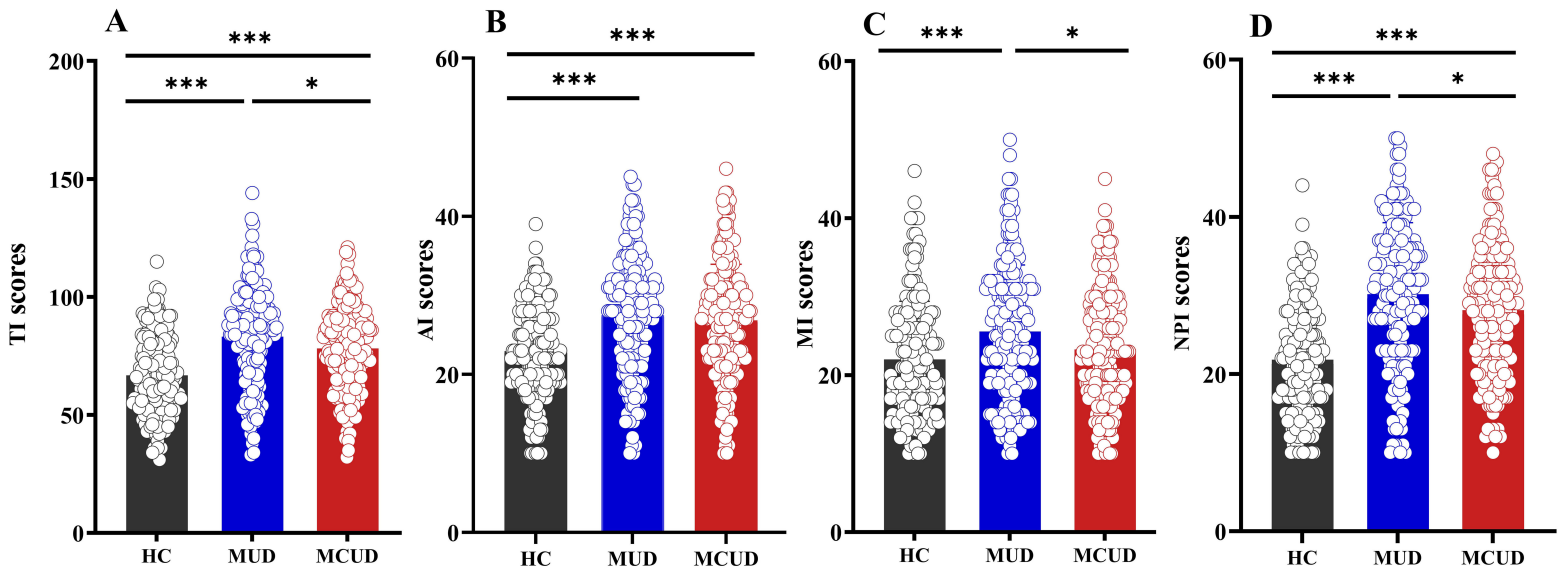
Differences in BIS among the three groups. **(A)** Comparison of total impulsivity scores among the three groups. **(B)** Comparison of attentional impulsivity scores among the three groups. **(C)** Comparison of motor impulsivity scores among the three groups. **(D)** Comparison of nonplanning impulsivity scores among the three groups. AI, attentional impulsivity; MI, motor impulsivity, NPI, nonplanning impulsivity, TI, total impulsivity. *p<0.05, **p<0.01, ***p<0.001.

*Post hoc* comparisons indicated that, for nonplanning impulsivity and total impulsivity, the differences between any two of the three groups were significant (**Figure 1**). With respect to attentional impulsivity, both the MUD group and the MCUD group had significantly higher scores than the HCs. For motor impulsivity, the MUD group had a significantly higher score than both the HCs and the MCUD group.

### 3.3 Differences in sensation seeking

For the SSS, owing to one missing data point in each of the HC and MCUD groups, the final participant count was adjusted to 205 in the MCUD group and 192 in the HC group. Age and years of education were also included as covariates in the ANOVA between groups.

**Figure 2:**
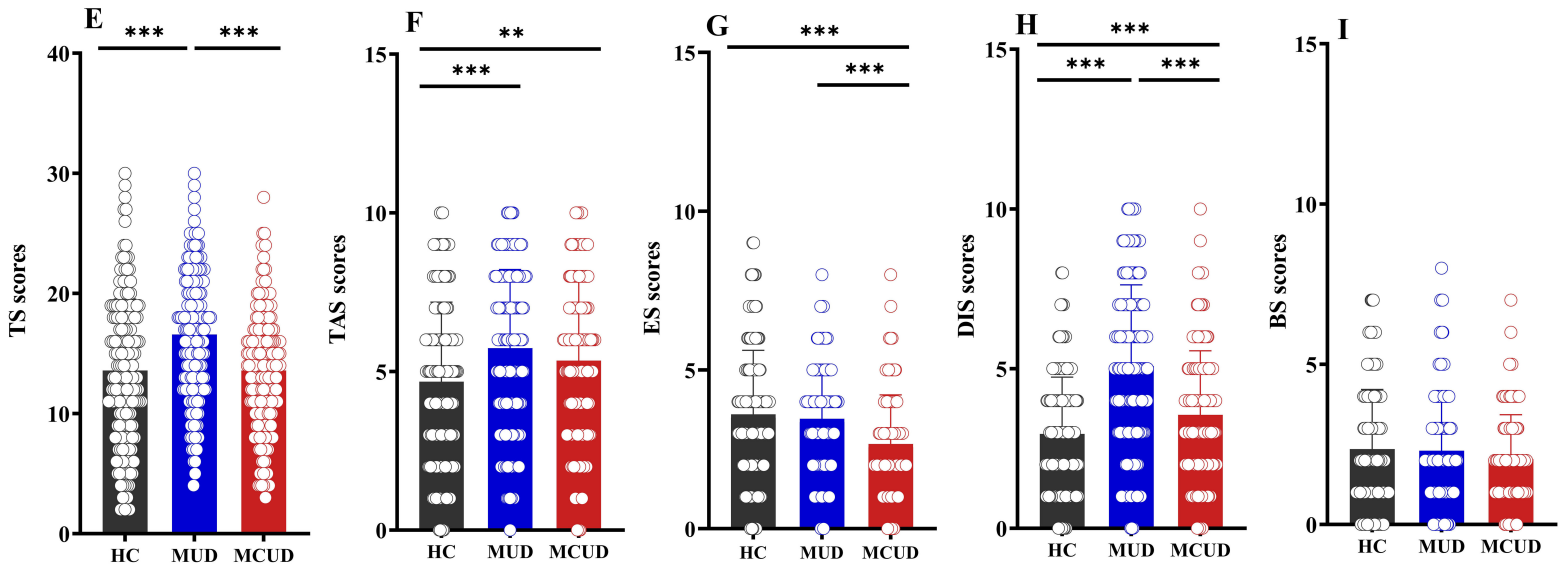
Differences in Sensation Seeking Scale among the three groups. **(E)** Comparison of total SSS scores among the three groups. **(F)** Comparison of thrill and adventure seeking scores among the three groups. **(G)** Comparison of experience seeking scores among the three groups. **(H)** Comparison of disinhibition scores among the three groups. **(I)** Comparison of boredom susceptibility scores among the three groups. TAS, thrill and adventure seeking; ES, experience seeking; DIS, disinhibition; BS, boredom susceptibility; TS, total SSS score. *p < 0.05; **p < 0.01; ***p < 0.001.

The analyses revealed that (as shown in **Table 2**) significant differences were observed among these three groups in thrill and adventure seeking, experience seeking, disinhibition, and the total score, but not in boredom susceptibility (*p* > 0.05). *Post hoc* comparisons indicated that the MUD group had significantly higher scores in thrill and adventure seeking, experience seeking, disinhibition, and total score than the HCs or the MCUD group (**Figure 2**). Compared with the HCs, the MCUD group had significantly higher scores for thrill and adventure seeking and for disinhibition.

### 3.4 Associations between impulsivity and sensation seeking

The correlation analyses revealed significant correlations between sensation seeking and the total BIS score (*r* = 0.20, *p* = 0.005) and motor impulsivity (*r* = 0.32, *p* < 0.001) within the MCUD group. The analyses also revealed significant correlations between sensation seeking and the total BIS score (*r* = 0.24, *p* = 0.001) as well as nonplanning (*r* = 0.19, *p* = 0.007) and motor impulsivity (*r* = 0.31, *p* < 0.001) within the MUD group. Among the HCs, sensation seeking was significantly correlated with the total BIS score (*r* = 0.15, *p* = 0.039) as well as motor impulsivity (*r* = 0.25, *p* < 0.001).

The correlation analyses also revealed that years of drug use were significantly correlated with the total BIS score (*r* = 0.19, *p* = 0.006) and sensation seeking score (*r* = 0.17, *p* = 0.018) within the MCUD group. However, years of drug use were not significantly correlated with the total BIS score or sensation seeking score within the MUD group.

### 3.5 Network estimation and comparison

**Figure 3:**
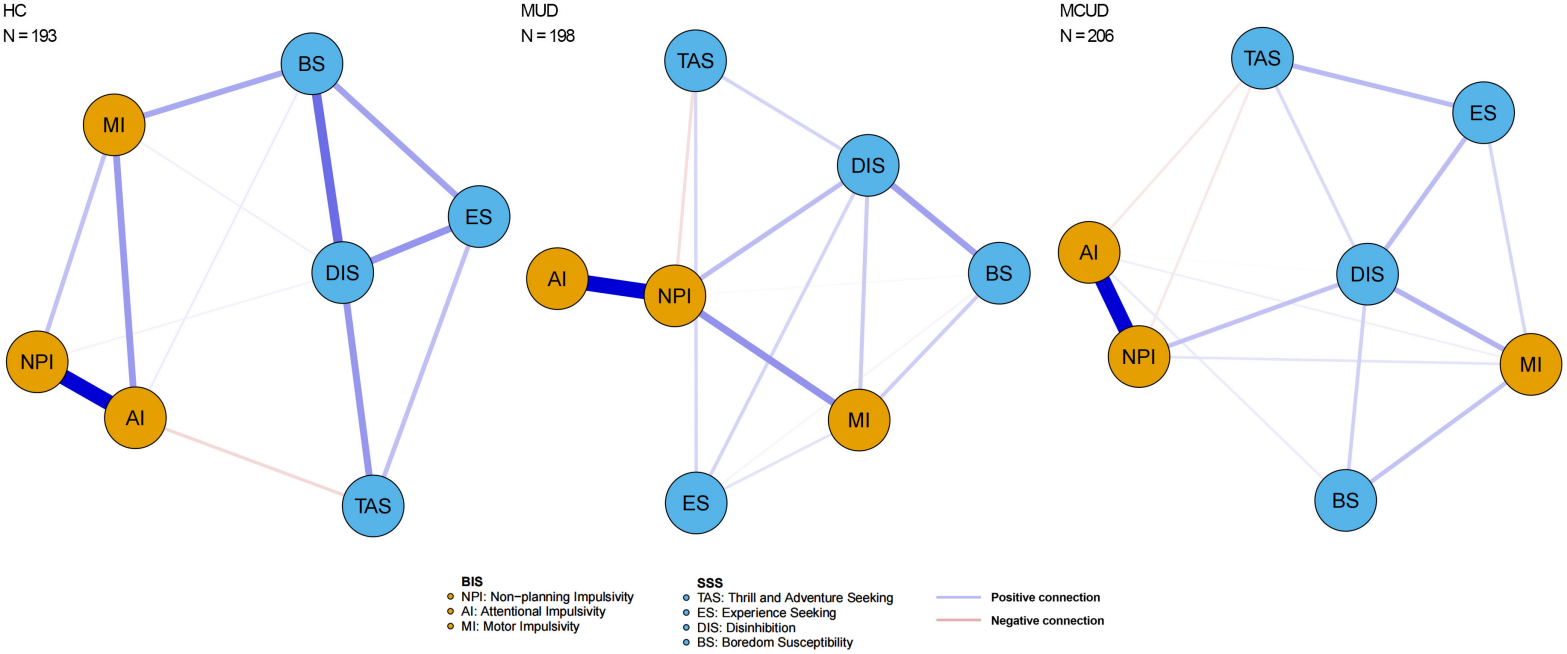
Network constructed by three dimensions in BIS and four dimensions in SSS of three groups.

**Figure 4:**
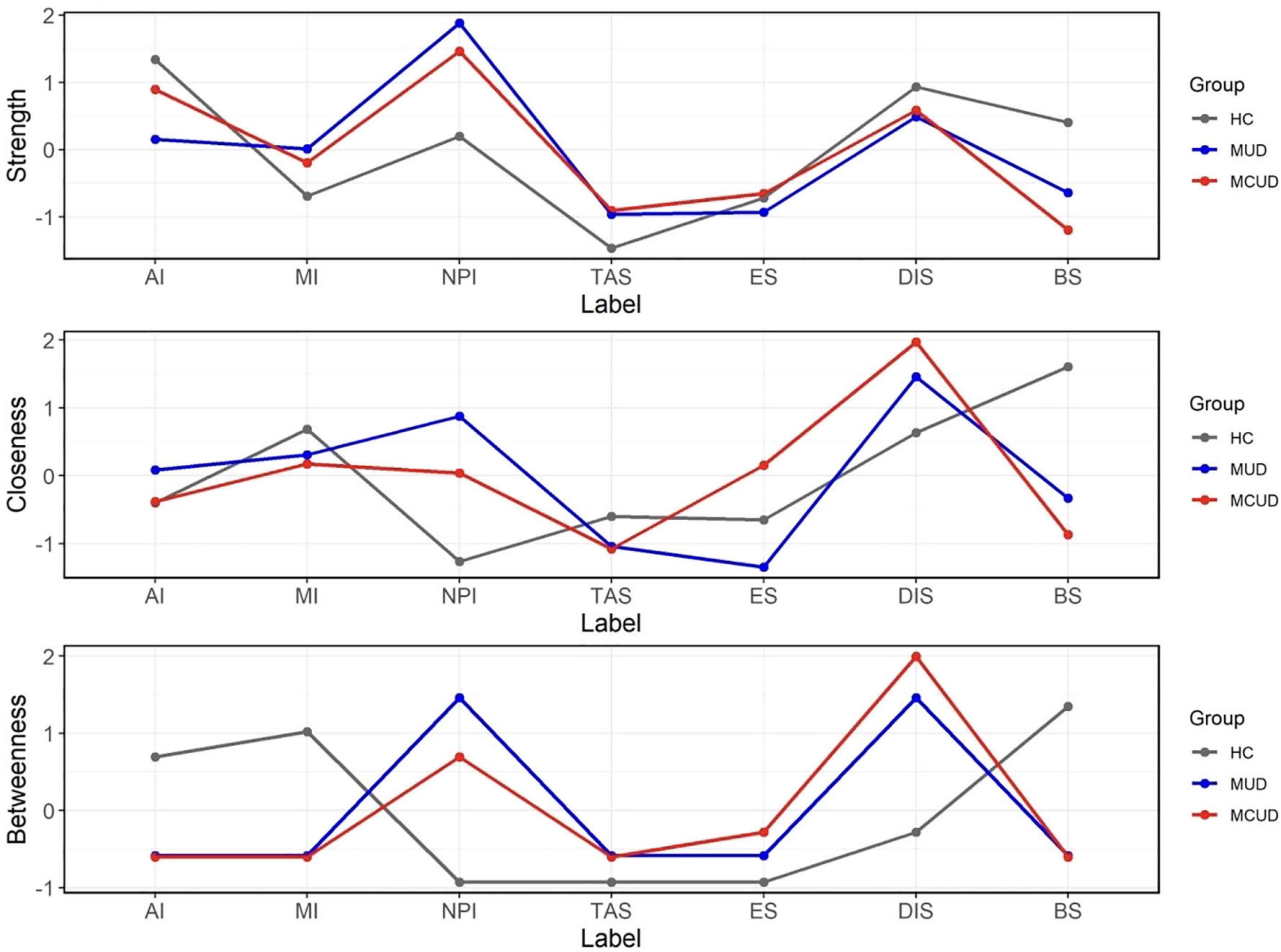
Centrality indices of each node in the three groups.

The trait impulsivity and sensation seeking networks of the three groups (MUD, MCUD, and HC) are shown in **Figure 3**. The node centrality indices of the three groups are shown in **Figure 4**, and the detailed centrality values of the three groups are displayed in **Supplementary Table S1**. Among both the MUD participants and the MCUD participants, the nonplanning impulsivity (NPI) exhibited the greatest strength, whereas disinhibition (DIS) presented the greatest closeness. In the MCUD group, DIS displayed the highest level of betweenness. Among the MUD participants, both DIS and NPI showed the highest betweenness.

The pairwise comparison of centrality indices among the three groups revealed that the participants in the HC group presented significantly greater betweenness centrality in the AI compared to those in the MUD group (diff = -5.00, *p* = 0.007, Bonferroni correction) and significantly higher closeness centrality in the BS compared to those in the MCUD group (diff = -0.011, *p* = 0.007, Bonferroni correction). Differences were not found in any centrality indices between the MUD and MCUD groups.

### 3.6 Differences in discount rate (*k*)

**Figure 5:**
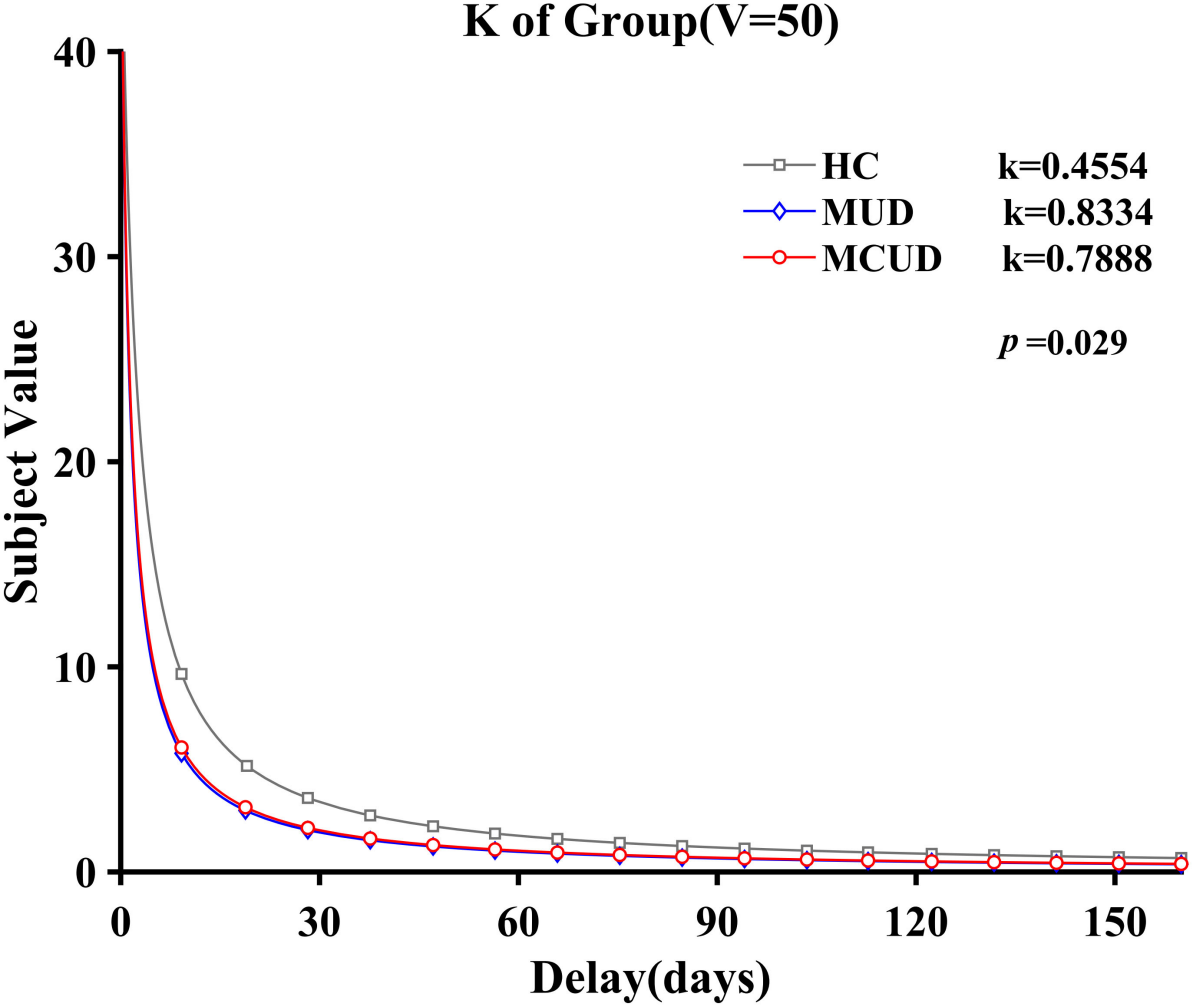
Relationship between subjective value and delays (*V* = 50 yuan).

Under the condition of an immediate reward of 50 yuan, *k*′ followed a normal distribution. The homogeneity of variance test indicated that the variances were homogeneous (*p* = 0.44). The ANOVA results revealed that the main effect of group was significant [*F*(2, 113) = 3.656, *p* = 0.029, *η* ^2^ = 0.061]. *Post hoc* analysis showed that the *k*′ values of the MCUD group (*M* = -0.94, SD = 1.63) and the MUD group (*M* = -0.96, SD = 1.61) were both significantly greater than those of the HCs (*M* = -1.82, SD = 1.69; *p* < 0.05). However, no significant differences were found between the *k*′ values of the MUD group and the MCUD group (see **Figure 5**).

**Figure 6:**
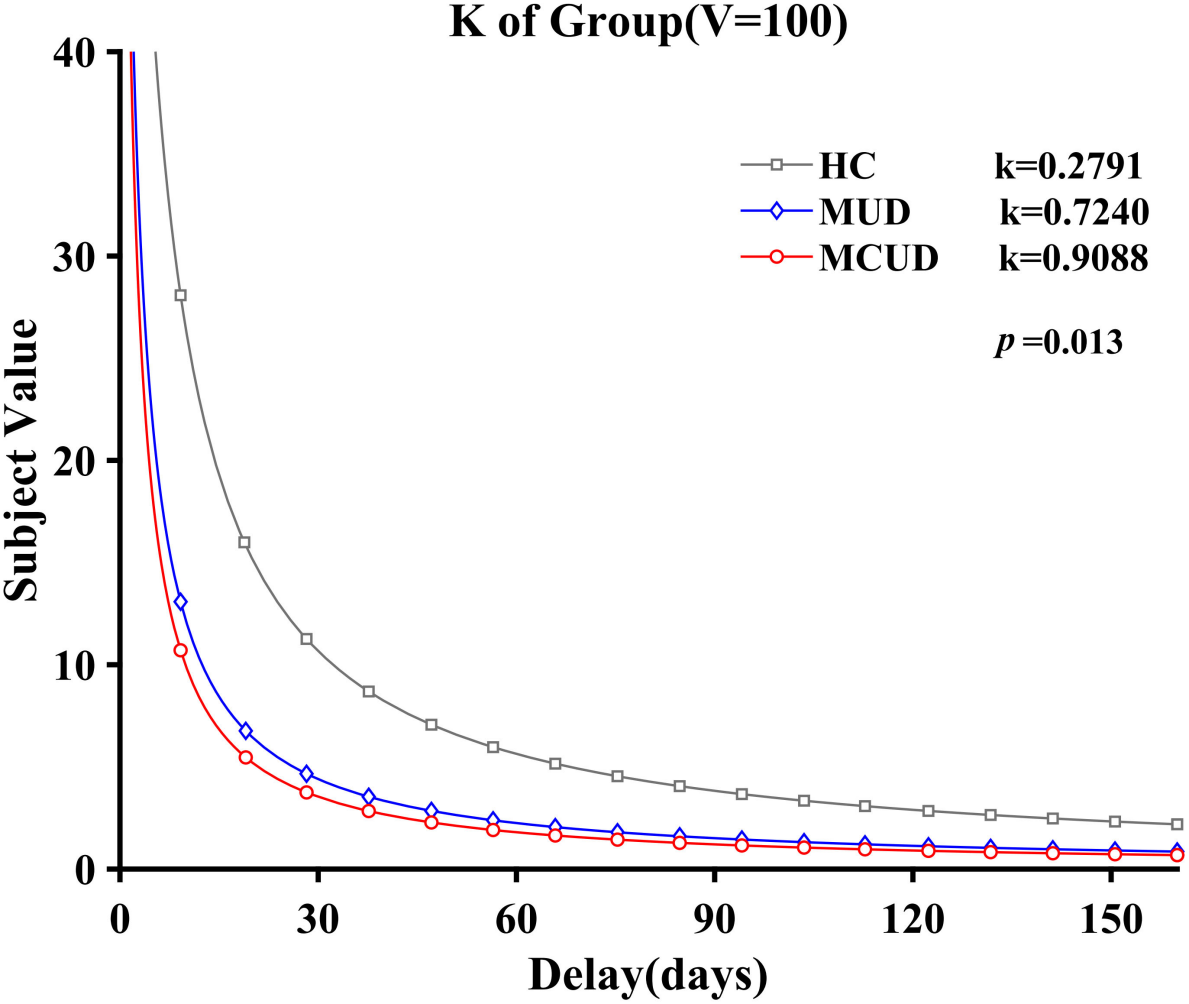
Relationship between subjective value and delays (*V* = 100 yuan).

Under the condition of an immediate reward of 100 yuan, *k*′ also followed a normal distribution. The homogeneity test confirmed variance homogeneity (*p* = 0.76). The analysis revealed significant differences among the three groups [*F*(2, 113) = 4.508, *p* = 0.013, *η* ^2^ = 0.074]. *Post hoc* analysis showed that the *k*′ values of the MCUD group (*M* = -1.34, SD = 1.94) and the MUD group (*M* = 1.57, SD = 2.00) were both significantly greater than those of the HCs (*M* = -2.62, SD = 2.08; *p* < 0.05). The results were similar to those obtained under the condition of 50 yuan (see **Figure 6**).

## 4 Discussion

The present study extensively examined differences in impulsivity between individuals with MUD and MCUD. With respect to impulsive personality traits measured by the BIS, individuals with MUD showed greater impulsivity than those with MCUD. For impulsive behavior measured by the delay discounting task, no significant differences were observed between the two addiction groups. However, they both exhibited greater impulsivity than the HC group. These results partly supported our hypotheses.

Self-reported impulsivity measured by the scale to some degree can reflect one’s personality traits and has been found to be associated with addictive behavior (7, 43, 44). Our results revealed increased impulsivity in individuals with MUD and MCUD compared to those with HCs, which is in accordance with the findings of previous studies (45). In addition, individuals with MUD had greater BIS impulsivity than those with MCUD, which is consistent with the findings of Zhang et al. (27). In fact, all participants in study 2 also completed the BIS. The findings of study 2 regarding impulsivity variations across the three groups replicated those from study 1, with significant differences emerging among the groups.

The SSS measures individuals’ risky behaviors. In terms of the total SSS score, individuals with MUD presented significantly greater levels of sensation seeking than both those with MCUD and healthy controls. This underscores the heightened tendency among MUD participants to engage in risky behaviors, distinguishing them from the other two groups, as confirmed in previous studies (46, 47).

The correlation analysis revealed a consistent positive correlation between sensation seeking and the total impulsivity score across all three groups, which was consistent with the findings of previous studies (48). However, across all three groups, we failed to find a significant relationship between sensation seeking and attentional impulsivity. This lack of correlation may have stemmed from the fundamental distinction between the constructs being measured: while the SSS primarily assesses behavioral tendencies toward novelty and excitement (49), attentional impulsivity primarily reflects cognitive impulsivity, which pertains to difficulties in controlling attention and inhibiting inappropriate responses.

To better understand the interplay between the two distinct traits across the three groups, we further conducted a network-based analysis, constructing individualized impulsivity and sensation seeking networks for each group. The strength centrality showed that nonplanning impulsivity (i.e., being unable to make plans before doing things) was the most important feature in both the MUD participants and the MCUD participants. The closeness centrality results revealed that disinhibition was the most important feature in both the MUD participants and the MCUD participants. The betweenness centrality revealed that disinhibition was the most important feature in individuals with MCUD, and both disinhibition and nonplanning impulsivity were the most important features in individuals with MUD. These results suggested that nonplanning and disinhibition were the core features of all the addiction groups—that is, they cannot make plans before acting or doing things as planned. A previous study suggested that disinhibition could be used to search for and identify adolescents with addictive tendencies (50). This finding also indicated that being relatively free from social constraints is a typical feature among addicted individuals. The pairwise comparison of centrality indices revealed that no significant differences were found in any of the centrality indices between the MUD and MCUD participants, but both groups differed significantly from the HCs.

The results of the DDT suggested that the addiction groups displayed impaired impulsive decision-making (significantly higher discount rates than did the healthy group), which is in line with the findings of previous studies (51, 52). The discount rate of MUD participants did not differ significantly from that of MCUD participants, suggesting similar levels of behavioral impulsivity between the two groups, which was inconsistent with the BIS results. Given that previous researchers have suggested that the impulsiveness scale and the DDT test different facets of impulsivity and that these factors are largely unrelated to each other (29, 53), the current findings appear to be both logical and consistent. In addition, we also conducted a correlation analysis between impulsivity and the delay discounting rate and failed to identify any significant correlation within any of the three groups. This result may also indirectly support the aforementioned research findings. However, it should also be noted that the specific setting and state of the participants might have influenced task performance at a particular time.

Overall, the results of this study indicated that both addiction groups exhibited significantly greater impulsivity than the control group, both in terms of trait and behavior. The difference between the MUD and MCUD participants was observed only in trait impulsivity and not in behavior. As a representative new psychoactive substance, methcathinone has many similarities with methamphetamine. As this study revealed, despite the differences in impulsivity questionnaires between individuals with MCUD and MUD, the network analysis showed that the two addiction groups share similar core features. This study further deepens our understanding of the characteristics of methcathinone and provides a reference for precise interventions for different drug users.

However, the current study has several limitations that should be considered. First, our participants were not random samples, and they were all male. Impulsive traits and behavior may differ between men and women; thus, the results of the present study may not necessarily be generalizable to both genders. Second, there were significant differences in the demographic variables among the three groups in the questionnaire study. Even though these variables were included as covariates in the ANOVA, we still cannot eliminate their influence. Third, the sample for the behavioral task was relatively small, which may have led to an insignificant difference between the two addiction groups in the DDT. However, a trend of difference was not observed, and the DDT results were therefore relatively credible. Nevertheless, the results of this study should be extrapolated with caution.

## 5 Conclusions

In conclusion, we found that the trait impulsivity of individuals with MUD was greater than that of individuals with MCUD. In contrast to our hypotheses, the impulsivity measured by the DDT of individuals with MUD was not greater than that of individuals with MCUD. However, for both measures of impulsivity, the two addiction groups scored higher than the controls. These results suggest that self-reported impulsivity and delay discounting test distinct aspects of impulsivity. The two aspects are interrelated and different. The present study explored differences in impulsivity among different individuals with drug use disorders and also further confirmed and expanded previous research on impulsivity.

## Data Availability

The raw data supporting the conclusions of this article will be made available by the authors without undue reservation.
