# Supplementary material for: Characterizing impulsivity in individuals with methamphetamine and methcathinone use disorders
Source: Front Psychiatry. 2024 Oct 16;15:1416342. doi: 10.3389/fpsyt.2024.1416342 (PMC11521857; doi:10.3389/fpsyt.2024.1416342)
Supplement: Supplementary file 1 [file Table1.docx]

Table S1. Detailed centrality values of the three groups

|  | strength | | |  | closeness | | |  | betweenness | | |
| --- | --- | --- | --- | --- | --- | --- | --- | --- | --- | --- | --- |
|  | MCUD | MUD | HC |  | MCUD | MUD | HC |  | MCUD | MUD | HC |
| AI | 0.895 | 0.156 | 1.339 |  | -0.383 | 0.083 | -0.403 |  | -0.603 | -0.586 | 0.696 |
| MI | -0.196 | 0.011 | -0.691 |  | 0.170 | 0.307 | 0.682 |  | -0.603 | -0.586 | 1.021 |
| NPI | 1.465 | 1.880 | 0.199 |  | 0.038 | 0.874 | -1.267 |  | 0.696 | 1.464 | -0.928 |
| TAS | -0.906 | -0.965 | -1.464 |  | -1.080 | -1.043 | -0.603 |  | -0.603 | -0.586 | -0.928 |
| ES | -0.650 | -0.932 | -0.718 |  | 0.156 | -1.347 | -0.650 |  | -0.278 | -0.586 | -0.928 |
| DIS | 0.587 | 0.487 | 0.931 |  | 1.968 | 1.457 | 0.637 |  | 1.996 | 1.464 | -0.278 |
| BS | -1.195 | -0.637 | 0.404 |  | -0.870 | -0.332 | 1.605 |  | -0.603 | -0.586 | 1.346 |

Note. AI= Attentional impulsivity, MI=Motor impulsivity, NPI=Nonplanning impulsivity; TAS=Thrill and Adventure Seeking, ES=Experience Seeking, DIS=Disinhibition, BS=Boredom Susceptibility.
